# Correction: A computational reproducibility study of PLOS ONE articles featuring longitudinal data analyses

**DOI:** 10.1371/journal.pone.0269047

**Published:** 2022-05-23

**Authors:** Heidi Seibold, Severin Czerny, Siona Decke, Roman Dieterle, Thomas Eder, Steffen Fohr, Nico Hahn, Rabea Hartmann, Christoph Heindl, Philipp Kopper, Dario Lepke, Verena Loidl, Maximilian Mandl, Sarah Musiol, Jessica Peter, Alexander Piehler, Elio Rojas, Stefanie Schmid, Hannah Schmidt, Melissa Schmoll, Lennart Schneider, Xiao-Yin To, Viet Tran, Antje Völker, Moritz Wagner, Joshua Wagner, Maria Waize, Hannah Wecker, Rui Yang, Simone Zellner, Malte Nalenz

There is an error in affiliation 5 for author Verena Loidl. The correct affiliation is: Institute for Medical Information Processing, Biometry, and Epidemiology—IBE, LMU Munich, Munich, Germany.
