# Supplementary material for: A computational reproducibility study of PLOS ONE articles featuring longitudinal data analyses
Source: PLoS One. 2021 Jun 21;16(6):e0251194. doi: 10.1371/journal.pone.0251194 (PMC8216542; doi:10.1371/journal.pone.0251194)
Supplement: Attachment — Submitted filename: 2019_08_response.pdf [file pone.0251194.s001.pdf]

Review and response to the registered report

PONE-D-19-08516R1

A computational reproducibility study of PLOS ONE articles featuring longitudinal data analyses

PLOS ONE

Dear Dr. Seibold,

Thank you for submitting your revised manuscript.

We sought further input from the adviser who reviewed the earlier version of the proposal as well as from the statistical reviewer, both have indicated that they find the changes incorporated satisfactory.

We have also reviewed the proposal internally and we identified a few areas where we feel further information or clarification is required to ensure that the research question addressed is clear and also that the methodology is reported in a way that makes the work fully reproducible, can we therefore ask you to please further revise the manuscript to address the items listed below:

- The manuscript outlines this as the aim of the project: 'Our aim with this study is to better understand the current practices in PLOS ONE papers dealing with longitudinal data'. This implies that the project will look at practices in PLOS ONE publications generally, which the methodology is not equipped to address given the small sample involved and the lack of information on whether the articles included are representative of longitudinal studies published in the journal. Can you please reframe the research question and aim to describe a project on reproducibility that involves a sample of longitudinal studies published in PLOS ONE, and avoid language that implies an assessment of practice or publications in PLOS ONE generally.

We updated to "Our aim with this study is to better understand the current practices in 11 PLOS ONE papers dealing with longitudinal data in terms of methodology applied but also in how results were computed and how it is made available for readers."

- Can you provide some more information on the selection process for the original group of 57 articles.

We added the following text: The PLOS website search function was utilized to scan through PLOS ONE published works. Key words used were "mixed model", "generalized estimating equations", "longitudinal study" and "cohort study".

- The project assigns one paper per student, the approach may result on potential confounding by inter-participant differences. There is also a risk that students with higher difficulty in reproducing the work may be more likely to drop out from the project. Please discuss these items further as part of the approach to implementation and limitations of the study.

None of the students dropped out of the course. Confounding is possible, but as we discuss in the article (see Section "Project circumstances") all students received help from the teachers. It is just as likely that there is confounding in that the researchers are more skilled in some topics than in others. We believe that any reproducibility study can suffer from these biases. The students probably spent much more time diving into the details of the paper than an individual reasearch could have done.

- You indicate two papers were excluded because 'Two authors prohibited to use their work within our study', given that the articles are published under a CC BY license reuse of the publicly available article and data should be possible, could you please nuance or revise this statement?

We added some additional text: We note that authors do not have the right to prohibit the reuse of their work as all papers are published under CC-BY license. However the negative

respones lead us to drop the papers, as we expected to have the need to contact authors with questions.

- Please provide further description of how 'success' of the replication will be assessed and established, i.e. what defines a successful replication, and how 'similar' results are defined.

We added a clear description now and incorporated this also in the tables.

Reviewers' comments:

Reviewer's Responses to Questions

Comments to the Author

1. If the authors have adequately addressed your comments raised in a previous round of review and you feel that this manuscript is now acceptable for publication, you may indicate that here to bypass the "Comments to the Author" section, enter your conflict of interest statement in the "Confidential to Editor" section, and submit your "Accept" recommendation.

Reviewer #1: All comments have been addressed

2. Is the manuscript technically sound, and do the data support the conclusions?

The manuscript must describe a technically sound piece of scientific research with data that supports the conclusions. Experiments must have been conducted rigorously, with appropriate controls, replication, and sample sizes. The conclusions must be drawn appropriately based on the data presented.

Reviewer #1: (No Response)

3. Has the statistical analysis been performed appropriately and rigorously?

Reviewer #1: (No Response)

4. Have the authors made all data underlying the findings in their manuscript fully available?

The PLOS Data policy requires authors to make all data underlying the findings described in their manuscript fully available without restriction, with rare exception (please refer to the Data Availability Statement in the manuscript PDF file). The data should be provided as part of the manuscript or its supporting information, or deposited to a public repository. For example, in addition to summary statistics, the data points behind means, medians and variance measures should be available. If there are restrictions on publicly sharing data—e.g. participant privacy or use of data from a third party—those must be specified.

Reviewer #1: (No Response)

5. Is the manuscript presented in an intelligible fashion and written in standard English?

PLOS ONE does not copyedit accepted manuscripts, so the language in submitted articles must be clear, correct, and unambiguous. Any typographical or grammatical errors should be corrected at revision, so please note any specific errors here.

Reviewer #1: (No Response)

6. Review Comments to the Author

Please use the space provided to explain your answers to the questions above. You may also include additional comments for the author, including concerns about dual publication, research ethics, or publication ethics. (Please upload your review as an attachment if it exceeds 20,000 characters)

Reviewer #1: (No Response)

7. PLOS authors have the option to publish the peer review history of their article (what does this mean?). If published, this will include your full peer review and any attached files.

If you choose “no”, your identity will remain anonymous but your review may still be made public.

Do you want your identity to be public for this peer review? For information about this choice, including consent withdrawal, please see our Privacy Policy.

Reviewer #1: No

[NOTE: If reviewer comments were submitted as an attachment file, they will be attached to this email and accessible via the submission site. Please log into your account, locate the manuscript record, and check for the action link “View Attachments”. If this link does not appear, there are no attachment files to be viewed.]

While revising your submission, please upload your figure files to the Preflight Analysis and Conversion Engine (PACE) digital diagnostic tool, https://pacev2.apexcovantage.com/. PACE helps ensure that figures meet PLOS requirements. To use PACE, you must first register as a user. Registration is free. Then, login and navigate to the UPLOAD tab, where you will find detailed instructions on how to use the tool. If you encounter any issues or have any questions when using PACE, please email us at figures@plos.org. Please note that Supporting Information files do not need this step.

In compliance with data protection regulations, you may request that we remove your personal registration details at any time. (Remove my information/details). Please contact the publication office if you have any questions.
