# Supplementary material for: A computational reproducibility study of PLOS ONE articles featuring longitudinal data analyses
Source: PLoS One. 2021 Jun 21;16(6):e0251194. doi: 10.1371/journal.pone.0251194 (PMC8216542; doi:10.1371/journal.pone.0251194)
Supplement: Attachment — Submitted filename: 2020_10_response.pdf [file pone.0251194.s002.pdf]

PONE-D-20-25993: A computational reproducibility study of PLOS ONE articles featuring longitudinal data analyses

PLOS ONE

Dear Dr. Seibold,

Thank you for submitting your manuscript to PLOS ONE. After careful consideration, we feel that it has merit but does not fully meet PLOS ONE's publication criteria as it currently stands. Therefore, we invite you to submit a revised version of the manuscript that addresses the points raised during the review process.

The two reviewers and I agree that your study was well done, clearly reported, and relevant, and that your work contributes to our understanding of reproducibility of studies using longitudinal analyses. Yet, several issues need to be dealt with in your revision.

First, the two reviewers requested some additional clarity of the type of submission (registered report or not). I apologize for not indicating to the reviewers that your work was originally submitted as a registered report but later handled as a standard submission. Please clarify this.

Second, both reviewers asked you to provide additional details of the methods, sampling, operationalizations, and data access. Both reviewers provided detailed feedback on the reporting and analyses that I ask you to consider as you revise your submission.

Third, the reviewers indicate that the main goals and main results could be presented more clearly in several sections of the manuscript. It is also import to be clear on your definition of reproducibility as you present the main results in the abstract.

Please submit your revised manuscript by Nov 16 2020 11:59PM. If you will need more time than this to complete your revisions, please reply to this message or contact the journal office at plosone@plos.org. When you're ready to submit your revision, log on to https://www.editorialmanager.com/pone/ and select the 'Submissions Needing Revision' folder to locate your manuscript file.

Please include the following items when submitting your revised manuscript:

- A rebuttal letter that responds to each point raised by the academic editor and reviewer(s). You should upload this letter as a separate file labeled 'Response to Reviewers'.
- A marked-up copy of your manuscript that highlights changes made to the original version. You should upload this as a separate file labeled 'Revised Manuscript with Track Changes'.
- An unmarked version of your revised paper without tracked changes. You should upload this as a separate file labeled 'Manuscript'.

If you would like to make changes to your financial disclosure, please include your updated statement in your cover letter. Guidelines for resubmitting your figure files are available below the reviewer comments at the end of this letter.

If applicable, we recommend that you deposit your laboratory protocols in protocols.io to enhance the reproducibility of your results. Protocols.io assigns your protocol its own identifier (DOI) so that it can be cited independently in the future. For instructions see: http://journals.plos.org/plosone/s/submission-guidelines#loc-laboratory-protocols

We look forward to receiving your revised manuscript.

Kind regards,

Jelte M. Wicherts Academic Editor PLOS ONE

Journal Requirements: When submitting your revision, we need you to address these additional requirements.

1. Please ensure that your manuscript meets PLOS ONE's style requirements, including those for file naming. The PLOS ONE style templates can be found at

https://journals.plos.org/plosone/s/file?id=wjVg/PLOSOne_formatting_sample_main_body.pdf and

https://journals.plos.org/plosone/s/file?id=ba62/PLOSOne_formatting_sample_title_authors_affiliations.pdf

Ok.

2. We suggest you thoroughly copyedit your manuscript for language usage, spelling, and grammar. If you do not know anyone who can help you do this, you may wish to consider employing a professional scientific editing service.

Upon resubmission, please provide the following:

- A marked-up copy of your manuscript that highlights changes made to the original version. You should upload this as a separate file labeled 'Revised Manuscript with Track Changes'.The name of the colleague or the details of the professional service that edited your manuscript
- A copy of your manuscript showing your changes by either highlighting them or using track changes (uploaded as a *supporting information* file)
- A clean copy of the edited manuscript (uploaded as the new *manuscript* file)

Ok.

3. In your manuscript text, we note that "We did not choose the papers randomly, but based on the set of potential papers given to us by PLOS ONE and then selected all papers meeting our criteria". In the Methods, please ensure that you have included the following:

- Information about how the initial set of 57 papers was selected, including any inclusion/exclusion criteria applied, so that other interested researchers can reproduce this analysis. We would also recommend that the complete set of 57 initial papers be provided as a supplementary information file.
- The complete inclusion/exclusion criteria used to select the initial set of 14 papers from the 57 papers that were identified.
- The complete inclusion/exclusion criteria used to select the final set of 11 papers.

Thank you. We updated the manuscript accordingly. Please let us know if we should move any further information from Figure 1 to the text.

4. Please amend the manuscript submission data (via Edit Submission) to include authors Severin Czerny, Siona Decke, Roman Dieterle, Thomas Eder, Steffen Fohr, Nico Hahn, Rabea Hartmann, Christoph Heindl, Philipp Kopper, Dario Lepke, Verena Loidl, Maximilian Mandl, Sarah Musiol, Jessica Peter, Alexander Piehler, Elio Rojas, Stefanie Schmid, Hannah Schmidt, Melissa Schmoll, Lennart Schneider, Xiao-Yin To, Viet Tran, Antje Volker, Moritz Wagner, Joshua Wagner, Maria Waize, Hannah Wecker, Rui Yang, Simone Zellner.

Done.

5. Review Comments to the Author

Thank you so much for your thorough and constructive feedback. We truly think that your input has improved the paper and have rarely seen such helpful reviews. We hope that we have answered all your questions to your satisfaction. Please let us know if we missed or misunderstood anything. All text changes are marked in blue.

Reviewer #1: Manuscript ID: PONE-D-20-25993 Manuscript title: A computational reproducibility study of PLOS ONE articles featuring longitudinal data analyses

Summary This article reports a retrospective observational study designed to test the analytic reproducibility of a small set of PLOS ONE articles containing longitudinal analyses. Specifically, as a part of a class exercise, the authors and their students attempted to repeat the original analyses performed in the

selected articles to see if they could obtain similar results. A range of difficulties reproducing the original results were encountered – some of which could be resolved through contact with the original authors. The generalizability of the results is quite limited due to the small sample size and somewhat ad-hoc sampling procedures; however, the authors appropriately calibrate their conclusions to the evidence, for example stating that "We can and should not draw conclusions from our findings on the 11 selected papers on the broader scientific landscape."

Generally, the paper is clearly written and is concise, though I think some important information is absent (see detailed comments below). The study appears to be transparently reported with analysis scripts and data for each reproducibility attempt made publicly available in an Open Science Framework repository. I only checked this repository superficially – one issue is that I could not seem to identify a data file for the study (see comment below) which needs to be addressed.

Important note: After reading the paper and writing my review, I was surprised to find a document included after the reference section with the title "Review and response to the registered report". I was not informed that this study was a registered report and as far as I can tell this is not mentioned in the manuscript aside from this appended document. Can the circumstances of this study be clarified? If this is a registered report then I feel I should be given more information. Most importantly, I need to see the original registered protocol. I would also like to see the prior review history and know whether the original stage 1 reviewers are also appraising the stage 2 manuscript.

We had initially submitted a registered report for this study to PLOS ONE, but since the review process took longer than the project, we decided – together with PLOS ONE editors – that it would be more scientifically sound to withdraw the submission and only submit the final paper. We submitted including the full history of reviews and hope you will receive the full information in the upcoming round of reviews.

Major Comments

- The exact operationalization of several of the outcome variables could be made clearer. For example, for the question "Are the methods used clearly documented in paper or supplementary material (e.g. analysis code)?" what was considered 'clear' vs. 'unclear'? For "Do we receive the same (or very similar) numbers in tables, figures and models?" what was considered 'similar' vs. 'dissimilar'? For "What are characteristics of papers which make reproducibility easy/possible or difficult/impossible?" – exactly what characteristics were examined?

We added a more detailed explanation what we considered a clear description in the methods section. As we only used R in our analysis we speak of a clear documentation, if it allowed us to follow the authors steps in a different software environment. As similar outputs we defined, if they have the same interpretation, as some deviations are expected. If already deviations in simple summary tables occured we immediatly contacted the authors, to get more information on the data. The characteristics examined were based on the discussion of the individual papers and what we identified as the most likely reasons for success/failures. We added a line to point this out.

- The oversight of the student work could be described in more detail – did teachers fully

We added two sentences which we hope will clarify the setup further: "Internal peer-reviews ensured that all groups had a solid technical setup. Finally all projects were carefully evaluated by the teachers and updated in case of problems."

- In the methods section, the sampling procedure is somewhat unclear – for example, "57 papers were initially screened. The PLOS website search function was utilized to scan through PLOS ONE published works. Key words used were "mixed model", "generalized estimating equations", "longitudinal study" and "cohort study". 14 papers fulfilled the criteria and were selected". Where does the number 57 come from? For the 14 papers – did they fulfill the search criteria? Or the criteria in Fig 1? Or both?

The phrasing was a bit unfortunate. Thank you for spotting this. We hope it is more clear now.

- It seems to me that the selection criteria will have heavily biased the results in favour of positive reproducibility outcomes – for example, only studies that had confirmed data availability were selected and only studies where authors replied to contact and were favourable to the reproducibility attempt were included. Because these factors probably influenced the results quite substantially, I'd suggest this bias is mentioned in key parts of the paper like the abstract and introduction.

Very good point. We included a sentence ("Inclusion criteria were the availability of data and author consent.") in the abstract and another ("Note that we only selected papers which provided data openly online and where authors agreed with being included in the study. We assume that this leads to a positive bias in the sense that other papers would be more difficult to reproduce.") in the introduction.

- I examined the OSF repository (https://osf.io/xqknz/) for this study and it was unclear to me where to find the data for this study (I could find data for the studies that the authors attempted to reproduce). Could clear instructions be provided on how to find and access the study data?

The data can either be found in the data folder in the respective paper folder (e.g. `papers/01_2017_Wagner/data`) or it is automatically downloaded in the provided code.

- Could it be clarified if all analyses reported in eligible papers were examined or just the subset pertaining to longitudinal analyses?

We focused on the longitudinal data analyses. We clarified this in the text.

- It is reported that for one article partial analysis code was available and for a second article the full analysis code was made available during email exchanges with the original authors. Its not clear whether all original authors were explicitly asked to make their code available – could this be clarified? If so, what were the responses to this query? Were reasonable justifications provided for not sharing the original code?

We are unsure in how much detail we are allowed to share the email exchanges openly. Aside from the initial contact, email exchanges were not standardized and authors were not asked if we would be allowed to share the details of the conversation. Not all authors were asked to share the code. We hope this answers your questions.

- The operational definition of reproducibility could be made clearer in the methods section (and perhaps also in the introduction) – in the results section the authors state "we define similar results as having the same implied interpretations" – this seems to be a less strict definition than used in other studies of analytic reproducibility (e.g., Hardwicke et al., 2018; 2020; Minocher et al., 2020). Some clarification and comment on this would be helpful for understanding the results.

Thank you for the interesting references. We agree that our operationalisation is less strict than others, such as the ones quoted by you. The main reason for this is, that we deliberately deviate from the software used by the authors, so deviation is to be expected to a certain degree and does not necessarily imply any kind of error on the side of the authors. However a detailed enough method description to be able to reach similar results, when using a different software implementation is in our eyes a minimal requirement for successful reporting. We added more explanation to the methods section.

- I think it would be informative to mention in the abstract how many analyses were reproducible only when assistance was provided by original authors.

Thank you for the suggestion, we added the number to the abstract.

- This sentence in the discussion is unclear – "We did not choose the papers randomly, but based on the set of potential papers given to us by PLOS ONE and then selected all papers meeting our criteria (see

Figure 1).” If the papers were given to the authors by PLOS ONE then this needs to be mentioned and explained at least in the methods section.

Thank you for spotting. We added the information in the methods section.

Minor Comments

- Terminology usage in this domain is diverse and sometimes contradictory (see e.g., https://doi.org/10.1126/scitranslmed.aaf5027) – I’d recommend including explicit definitions and avoiding use of loaded terminology if possible. For example, it would be good to have a clear definition of ‘computational reproducibility’ in the opening paragraph. The authors may also want to consider using the term ‘analytic reproducibility’ instead of computational reproducibility. Researchers in this domain have recently started to draw a distinction between the two concepts and the former seems more applicable to what the present study has addressed. The distinction is discussed in this article - https://doi.org/10.31222/osf.io/h35wt – specifically, “Computational reproducibility is often assessed by attempting to re-run original computational code and can therefore fail if original code is unavailable or non-functioning (e.g., Stodden et al., 2018; Obels et al., 2019). By contrast, analytic reproducibility is assessed by attempting to repeat the original analysis procedures, which can involve implementing those procedures in new code if necessary (e.g., Hardwicke et al., 2018; Minocher et al., 2020)”

Thank you very much for hinting us to the article by Hardwicke et al. This is indeed highly relevant to our study as their setup is similar to ours. Furthermore we now mention the term analytic reproducibility and cite LeBel et al.

- An additional point on terminology - use of the term ‘replication’ (e.g., in the abstract and introduction) should perhaps be avoided if possible in this context because it is often used to mean “repeating original study methods and obtaining new data” – whereas here it is being used synonymously with computational reproducibility to mean “repeating original study analyses on the original data” (see http://arxiv.org/abs/1802.03311)

You are completely right. We believe this increases clarity in our manuscript. Thank you!

- I felt the study design could be made much more explicit in the introduction. For example, “The articles we chose are [1–11]” – briefly mentioning the sampling procedure would be helpful here so the reader can understand the study design (e.g., was it a random sample, arbitrary sample, etc).

We added the sentence “They are all PLOS ONE papers which fulfilled our selection criteria [. . .] in March 2019.”

- The rationale for the study could be made clearer in the introduction. The review of existing literature in this domain is sparse – it is not clear what knowledge gap the study is trying to fill. How does this work build on previous studies and/or extant knowledge in this domain? Why focus on these 11 papers? Why focus on PLOS ONE?

Thank you. This is a very important question which we now answer in the introduction.

- It would be helpful to define acronyms e.g., what is a “6 ECTS course”?

Thanks for spotting. ECTS are credit points according to the European Credit Transfer and Accumulation System. We updated the sentence.

- This is unclear and perhaps needs rewording: “For problems with implementation specifics for methods described in the papers”

We agree. We did so and hope it is more understandable now: “Students were advised to contact the authors directly in case of unclear specifications of methods.”

- “RequirementR.3 is important to be able to contact the authors.” – this appears to just be a restatement of the requirement rather than a justification for including it.

Thanks for spotting all these small yet important things. We updated this.

- To ensure the reproducibility of their own analyses, the authors may wish to consider saving and sharing the computational environment in which the analyses were performed. Various tools are available to achieve this e.g., Docker, Binder, Code Ocean.

Some of the computations run very long and require server usage which makes it difficult to use the suggested solutions (our University servers do not allow usage of Docker). As we are of course aware of the issue, we decided to provide the `SessionInfo()` for each paper instead. We realized that this information is not provided in the paper and we added it (see Compuational Details).

Reviewer #2: What did they do The authors tried to reproduce 11 statistical analyses published in PLOS ONE that used longitudinal data. This was done by cleverly making use of student labor in the light of a university course. For each paper, a detailed summary file on the OSF describes the study, the model, the analyses, and potential deviations in results. Those files further contain the used R code allowing the verification of this reproducibility study (Personally, I did not make use of that possibility!).

General remarks I believe this work to be an important contribution to open science and a service to the scientific community in general. The manuscript is well-written and the authors delightfully refrained from being unnecessarily complicated. To put this work into perspective with similar empirical work on reproducibility in psychology, I suggest giving a more detailed description of methods, results, and, implications in the main manuscript. As of now, I am not sure which conclusions to reach about the state of reproducibility in PLOS one. A more detailed summary of the findings is particularly important in this case because each summary was written by a different teams of students making it very time-consuming to extract all the important information.

Major remarks

- I am confused as to the nature of this manuscript. Does it constitute a registered report? If so, the manuscript should clearly indicate what part of the work was done prior to the submission of the registered report and what was done afterward.

We had initially submitted a registered report for this study to PLOS ONE, but since the review process took longer than the project, we decided – together with PLOS ONE editors – that it would be more scientifically sound to withdraw the submission and only submit the final paper. We submitted including the full history of reviews and hope you will receive the full information in the upcoming round of reviews.

- I am missing a (short) Method section where you describe the timeline of the conducted reproductions (when where authors contacted to provide code of analysis?, how did the students work on the assignment?, how (much) assistance did they receive from the teaching team?) In line 340 an internal peer review is mentioned – please provide more information on that.

Thank you. We incorporated this suggestion.

- I agree with what is being said in lines 208-212, however, I would like to have precise information about when the magnitude of the effect is the same. Further, the possibility of achieving the same numbers by deliberately deviating from the method description of the paper should be discussed as this has implications on the implied interpretations.

We agree and added such comments in the discussion of the individual papers, when the difference was not negligible. In general we only deviated from the method description when it was unclear how to proceed, even after contacting the authors. In some cases, we also performed sensitivity analyses and mention the results, but they are not used to judge reproducibility.

- Roughly, we can group reproduction failures into 3 groups: Reporting errors in the paper, insufficient/incorrect description of methods or data that prevent reproduction, and software/algorithm differences. I would like to know for each reproduction failure the group to which it likely belongs.

Since you exclusively used R in your reanalyses whereas only 1 of the 11 papers did so, I think it is important to discuss software differences (including differences in algorithms, default/starting values..) in detail. Whereas software differences are negligible for simple designs as ANOVA and t-tests, this cannot just be assumed for GEEs and GLMMs. A discussion of software differences is, for example, important to interpret the results for paper #1 (lines 225 to 234) and also line 257. Looking at your summary file of this paper "essay_01.pdf" it turns out that you have deviated in multiple ways for a multitude of reasons from what was described in the paper. As a result, it is hard to judge whether the original analysis contains reporting errors or not. your analysis of paper #1 A related issue is when you apply a different optimization algorithm. It might be of interest to try to reproduce those papers where the reproduction attempt was unsuccessful (and where the provided data does not seem to be the culprit) via the software package (and the functions therein) used by the respective authors.

We agree with this insightful comment. In this study, software/algorithm differences are expected, as we use different software as most of the original studies. Due to this, differences in coefficients, especially in complex models, are expected to occur. We cannot differentiate between reporting errors and errors due to different implementations. The only errors, that we can spot with reasonable confidence are if the methods and data description are not clear enough for us to reach similar results using different implementations. This means, that starting values and other parameters used in the optimization need to be provided, if they can have a high impact on the results. If they were not provided, we relied on the standard settings in the respective R-libraries and reasonable trial and error. So for this study, we can only answer the question "can we reach the same interpretations following the authors descriptions but using different implementations". This is less strict than other operationalisations of reproducibility, but in our opinion reflects a very common situation for researchers. We added a column to the results table, that consists of the most likely reason for failures of reproduction for parts or all results.

- 233 – If you believe that your R code does not converge properly, it should be changed until it does, no? If you are unable to fit the model in R, it cannot be judged whether the published results are approximately correct or not. Now, all we know is that the students assigned to this paper were unable to properly fit the statistical model to the data via R.

Thank you for your comment. We did this study under the assumption of having access to only free software. We believe that it is too much to ask from someone aiming to reproduce a paper to write new software. Using licensed products comes with the possibility of using methods that are not openly available at all, which was the case here. We added more information on this in the manuscript.

- 316 – I would mention that in the abstract. The current abstract might give an incorrect impression as it is nowhere mentioned that the stated results involved author assistance (ideally, reproducibility in an open data journal should be possible without contacting the authors!)

Thanks for spotting this. We completely agree and added the sentence "For all but two articles we had to contact the authors to reproduce results."

Minor remarks

- 4 – use reproduction instead of replication. More generally, I suggest to use reproduce/reproducibility to describe computational reproducibility and replicated/replicability for new studies involving different data as this terminology is most commonly used in Psychology nowadays.

You are completely right. We believe this increases clarity in our manuscript. Thank you!

- 12 - Longitudinal data includes variables that are measured repeatedly over time but those variables do not necessarily have to do with humans.

Done, thanks.

- 111 – I would choose a more descriptive figure caption.

Done, thanks.

- 112 – I would refer to R.1 in singular (i.e. requirement R1)

Done, thanks.

- 130 – Is there the possibility of including additional papers? If so, I would like to see reproduction attempts of the 2 papers were the authors "prohibited" the use of their work to be reproduced.

We deliberately chose not to work with papers where the authors are not ok with us reproducing the paper as it did not fit in the context of our study. We hope you understand.

- 139-143 – Did you try to reproduce ALL figures and numbers reported in the paper that were related to the longitudinal study? If not, what was omitted and why? Please add 1 or 2 clarification sentences.

Thank you for finding this missing information. We added "If many analyses were performed, we focused on the analyses of longitudinal data."

- 144 – Reproducing someone else's analysis typically involves many RDFs, yes. But, it does not make sense to say (line 145) that there were many decisions that would adhere to YOUR steps 1-4. Instead, you should write that there are multiple ways to read in/process/analyze the provided data that are not in disagreement with what is stated in the paper or the supplementary material.

You are absolutely right. We updated the text.

- 147 – Please be more specific.

We added more context and hope it is more understandable now.

- 153 – The title is not self-explanatory, especially because it is written in the present tense. Maybe "Which statistical methods were used by the papers" instead.

Good point. We updated the caption.

- 162 – "according to statisticians" I would refrain from using such a phrase. Instead, just cite relevant papers arguing for GLMMs over GEEs and, potentially, summarize some of their advantages.

Thank you. Now that I read it again, it sounds funny to me, too. We updated the sentence.

- 172 – See comments for 153 above

Good point. We updated the caption.

- 184 – Did you always ask the authors for their source code. If not, when (before or after the 1st reproduction attempt?) did you ask for it. You provide some information in lines 201 to 207, but, I would like to know the specific time-line, and I want to know what was planned a-priori and what was ad hoc.

We did attempt to do everything on our own. Only if necessary information was not available, we contacted the authors. We are now making this more clear in the text.

- 247 – Where is the search procedure mentioned?

We updated the description to make it more clear how the search procedure was conducted: "The PLOS website search function was utilized to scan through PLOS ONE published works. Key words used were . . . "

- I would like to see the implications of the non-reproducible findings discussed. How many unreasonable original analyses (& conclusions drawn from it) could be identified? I know that this type of finger-pointing is uncomfortable, especially since only work from authors that provided both their data and responded to your e-mails were included in your sample, yet, it is important to estimate the rate of reporting errors and irreproducible findings in Psychology.

Out of the papers, that were not reproducible at all for us, in one case we were unable to preprocess the data, in one the failure was clearly due to implementation differences and only

in one case we identified a potentially unreasonable analysis. Of course for the two papers mentioned previously we just cant know, as we were unable to reach results ourselfs.

- The updated outline of the aim of this study is "Our aim with this study is to better understand the current practices in 11 PLOS ONE papers dealing with longitudinal data in terms of methodology applied but also in how results were computed and how it is made available for readers." I find this unnecessary complicated and, more importantly, it does not reflect the content of your study well at all. Wasn't the aim of this study to assess the extent to which papers analyzing longitudinal data in PLOS ONE could be reproduced by independent researchers.

You are right, thanks. We updated the section.
