# Supplementary material for: A computational reproducibility study of PLOS ONE articles featuring longitudinal data analyses
Source: PLoS One. 2021 Jun 21;16(6):e0251194. doi: 10.1371/journal.pone.0251194 (PMC8216542; doi:10.1371/journal.pone.0251194)
Supplement: Attachment — Submitted filename: 2021_03_response.pdf [file pone.0251194.s003.pdf]

PONE-D-20-25993R1: A computational reproducibility study of PLOS ONE articles featuring longitudinal data analyses

We thank the Editor and Reviewer for their support and comments. It has been a pleasure working with you so far and we hope that we were able to address all remaining issues. We have submitted an unmarked and a marked version of the manuscript (green for new changes, blue for changes in the previous round).

Since the previous submission we've added the following references: Obels et al 2020, Maassen et al 2020, and Artner et al 2020. Also we updated the citation to Hardwicke et al 2020 (we used to cite the preprint but it has been published in Royal Society Open Science in the meantime).

Review Comments to the Author Reviewer #2:

I find that this revised paper well-structured and easy to read, and I can recommend its publication as new insights on reproducibility are much-needed. What I would like to see added is a more thorough discussion of the state of reproducibility in PLOS ONE (as well as more generally in psychology) together with its implications (both in the introduction and the discussion section). In particular, this paper should include references to all relevant literature on this topic (see minor remarks below). Otherwise, a reader of this paper will not be made aware of other important empirical findings on this topic.

Thank you so much Mr Artner for your insightful review and constructive feedback. Please see our response to each point below. New changes in the manuscript are marked in green.

Below some minor remarks hopefully further improve the quality of this paper.

- Abstract: The last sentence only states quite obvious things. I would prefer to read about non-obvious insights gained in light of this study.

Thanks for this suggestion. As per the *writing in the sciences* course (see https://youtu.be/xmzUQ46YFiE), we added a sentence on the implications of our findings.

- Line 4: please include important recent studies on reproducibility such as
  - Artner, R., Verliefde, T., Steegen, S., Gomes, S., Traets, F., Tuerlinckx, F., & Vanpaemel, W. (2020). The reproducibility of statistical results in psychological research: An investigation using unpublished raw data. Psychological Methods.
  - Maassen, E., van Assen, M. A., Nuijten, M. B., Olsson-Collentine, A., & Wicherts, J. M. (2020). Reproducibility of individual effect sizes in meta-analyses in psychology. PloS one, 15(5), e0233107.
  - Obels, P., Lakens, D., Coles, N. A., Gottfried, J., & Green, S. A. (2020). Analysis of open data and computational reproducibility in registered reports in psychology. Advances in Methods and Practices in Psychological Science, 3(2), 229-237.

Thank you so much for sharing these interesting articles with us! I devoured them and gained many insights on what we ourselves could have done differently :). We included all suggested references and made some changes to the manuscript text.

- Line 33: What did other empirical investigations on reproducibility find?

I am not sure if that answers the question but we are writing about this in the first paragraph in the introduction. See lines 2-9.

- Lines 160-162: I would not label a lack of knowledge of the exact calculations performed by the authors due to a lack of information provided as RDFs. RDFs are what the original authors had! Maybe you can write: „The description about all these steps was generelly vague (see classification of reported results in Artner et. al, 2020) meaning that there were multiple ways in line with the descriptions in the originial paper. This study, thus, exposed a large amount of Researcher degrees of freedom [23] coupled with a lack in transparancy about in the original studies."

That is a great suggestion and now thinking about it more, I completely agree with you. We changed the text according to your suggestion (with a few tweaks). Thank you!

- I find the style in which the results section is written weird (until line 251). Why not just describe the results with a reference to the respective table. Now we have Tables first and it is not really clear if the text re-iterates the information in the tables or if additional information is provided. Also, why not merge tables 1, 2 and 3? Table 1 alone does not provide enough information to be included in the main text. Maybe the results section can be structured as follows: 1

Each paper has its own story and reasons why it was or wasn't reproducible and what the barriers were. With the individual sections we want to show how diverse the papers were and what types of challenges we faced. We now clarify this in the paper. We use 3 tables instead of one because the table would be too big for a PDF otherwise.

- Line 239: When was the magnitude considered to be the same? It is important to exactly describe your criteria here to allow the reader to gauge the overall results of your study. Without knowing whether your criterion was lenient or rather strict, it cannot be done as each and every one of us uses individual metrics to gauge similarity.

Very good point. We use a rather lenient definition in the result tables, but describe each result in more detail, to highlight problems or borderline cases. A formal definition is difficult, as the analyzed models are very different and it is hard to define a single criterion, that captures the results adequately. We believe that our lenient definition, together with the descriptive part works well in this smaller scale study, but probably would be impossible for large scale studies on reproducibility.

- Fig 2: General comment - Without knowing the range of parameter values it is hard to interpret differences in original and reproduced results. Why did you choose to report on the estimates of this article. Why not report, for example, on the results of article [1] instead?

This is a very good point. The plot shows regression coefficients from the fitted GEE model. We added a clarification to the paper. We chose to report this article as an example for our definition.

- Lines 261-264: Large differences in magnitude should result in different interpretation even in case of equal signs!!!!!

We agree that our conclusion was inconsistent in this case and changed the classification of article [1] to be not reproducible. In the other, reproducible, papers the deviations of coefficients were much smaller.

- Line 299: substantial!

Thanks for spotting!

- Line 351: Nevertheless we were only able . . . .

Thanks for spotting!
